# YM155 Inhibits NleB and SseK Arginine Glycosyltransferase Activity

**DOI:** 10.3390/pathogens10020253

**Published:** 2021-02-23

**Authors:** Congrui Zhu, Samir El Qaidi, Peter McDonald, Anuradha Roy, Philip R. Hardwidge

**Affiliations:** 1 College of Veterinary Medicine, Kansas State University, Manhattan, KS 66506, USA; congruiz@vet.k-state.edu (C.Z.); elqaidi@vet.k-state.edu (S.E.Q.); 2 HTS Laboratory, University of Kansas, Lawrence, KS 66047, USA; petemcd@ku.edu (P.M.); anuroy@ku.edu (A.R.)

**Keywords:** type three secretion system effectors, glycosyltransferase, enteric bacteria

## Abstract

The type III secretion system effector proteins NleB and SseK are glycosyltransferases that glycosylate protein substrates on arginine residues. We conducted high-throughput screening assays on 42,498 compounds to identify NleB/SseK inhibitors. Such small molecules may be useful as mechanistic probes and may have utility in the eventual development of anti-virulence therapies against enteric bacterial pathogens. We observed that YM155 (sepantronium bromide) inhibits the activity of *Escherichia coli* NleB1, *Citrobacter rodentium* NleB, and both *Salmonella enterica* SseK1 and SseK2. YM155 was not toxic to mammalian cells, nor did it show cross-reactivity with the mammalian O-linked N-acetylglucosaminyltransferase (OGT). YM155 reduced *Salmonella* survival in mouse macrophage-like cells but had no direct impact on bacterial growth rates, suggesting YM155 may have utility as a potential anti-virulence inhibitor.

## 1. Introduction

Enteric bacterial pathogens are important threats to human health and are major sources of foodborne disease. Enterohemorrhagic *E. coli* (EHEC) are especially important bacterial pathogens because they cause a type of renal failure (hemolytic uremic syndrome; HUS) for which therapies are limited [1]. A related attaching/effacing (A/E) pathogen, enteropathogenic *E. coli* (EPEC), is an important cause of infantile diarrhea. Recent EHEC outbreaks (e.g., O104:H4) have involved a combination of virulence traits characteristic of EHEC and of enteroaggregative *E. coli* (EAEC; [2]). *Salmonella* is also a leading cause of foodborne illnesses [3]. Understanding how these bacteria evade the mammalian immune system and develop small molecules to subvert these bacterial strategies is an important area of investigation.

We and others have characterized a conserved family of type III secretion system (T3SS) effector proteins that inhibits innate immune responses to infection [4,5,6,7,8]. These proteins (named NleB1 in EHEC and EPEC, NleB in *Citrobacter rodentum*, and SseK in *Salmonella enterica*) are glycosyltransferases that glycosylate host protein substrates with β-*D*-N-acetylglucosamine (GlcNAc) on arginine residues. These substrates include receptor-interacting serine/threonine-protein kinase 1 (RIPK1), tumor necrosis factor receptor (TNFR) type 1-associated DEATH domain protein (TRADD), and the Fas-associated protein with death domain (FADD). Some NleB/SseK orthologs also glycosylate glyceraldehyde 3-phosphate dehydrogenase (GAPDH) to reduce GAPDH binding to TRAF2 and inhibit NF-κB signaling [5].

Arginine glycosylation occurs on the guanidinium groups of arginines, which are poor nucleophiles. This modification is biologically important because the glycosylation of arginines on host protein substrates leads to target protein inactivation and disrupts the innate immune response. Mammals do not add N-GlcNAc residues to arginine, while this modification is important to *E. coli* and *Salmonella* virulence. Thus, inhibitors that prevent the formation of this unusual post-translational modification represent a potentially novel mechanism by which to combat infections. 

We previously developed and optimized a high-throughput screening (HTS) assay for NleB1 inhibitors. This assay produces a luminescent signal when UDP-GlcNAc is hydrolyzed by NleB1 to liberate UDP [9]. We showed from an initial screen of 5160 compounds from the University of Kansas Center of Excellence in Chemical Methodologies and Library Development library that two compounds, 100066N and 102644N, both inhibited NleB1, SseK1, and SseK2 activities [9]. The addition of these compounds to cultured mammalian cells inhibited NleB1 glycosylation of TRADD. These compounds were also capable of inhibiting *Salmonella* replication in mouse macrophage-like cells. Neither inhibitor was significantly toxic to mammalian cells, nor showed in vitro cross-reactivity with the mammalian O-linked N-acetylglucosaminyltransferase (OGT). 

The utility of 100066N and 102644N was limited because of the lack of their commercial availability and poor solubility. We were therefore motivated to screen a larger library of compounds to identify more diverse scaffolds that may be amenable to future chemical optimization. Here, we report the results of a 42,498 compounds screening assay, as well as the preliminary characterization of the activity of sepantronium bromide (YM155) against NleB/SseK.

## 2. Results

### 2.1. NleB1 Inhibitor Screening

We used a previously described HTS assay for NleB1 inhibitors that produces a luminescent signal when UDP-GlcNAc is hydrolyzed by NleB1 to liberate UDP [9]. The assay was performed by incubating purified recombinant NleB1 (150 nM) with UDP-GlcNAc (300 µM) for 2 h at 30 °C, followed by conversion of UDP to ATP, which is then utilized by luciferase to generate light in proportion to the UDP concentration. 

We screened 42,498 compounds (20 µM) derived from the University of Kansas Infectious Diseases Assay Development (KU-IDAD) laboratory from diversity sets representing a diverse scaffold collection, including Analyticon Natural products library (n = 4197), ChemDiv 3D Biodiversity (n = 19,528), ChemDiv Peptidomimetics (n = 12,139), FDA (n = 1280), Selleck Bioactives (n = 3354), and MayBridge mini (n = 2000) for their ability to inhibit NleB1 activity. 

**Figure 1:**
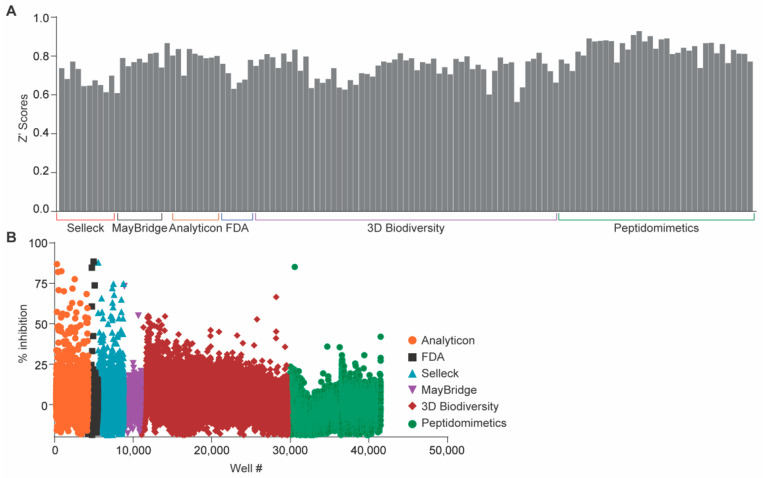
Primary screening results. (**A**) Distribution of Z’ scores across all plates in NleB1 inhibition assays. An average z’-score of 0.77 +/− 0.08 was obtained, reflecting good separation between the distributions of the positive and negative controls among all assay plates. (**B**) High-throughput screening (HTS) results. The percentage of inhibition was calculated by computing the luminescence signals from UDP liberated from NleB activity. Inhibition was then plotted as a function of each compound per well number for all 42,498 targets in the combined libraries.

Overall, we obtained a 0.47% hit rate (200 compounds) with an average z’-score of 0.77 +/− 0.08 (Figure 1A). Among the 200 compounds that significantly inhibited NleB1 activity, the hits were distributed among the libraries as follows: Analyticon Natural products library (n = 76), ChemDiv 3D Biodiversity (n = 59), ChemDiv Peptidomimetics (n = 8), FDA (n = 25), Selleck Bioactives (n = 28), and MayBridge mini (n = 4) (Figure 1B). 

**Figure 2:**
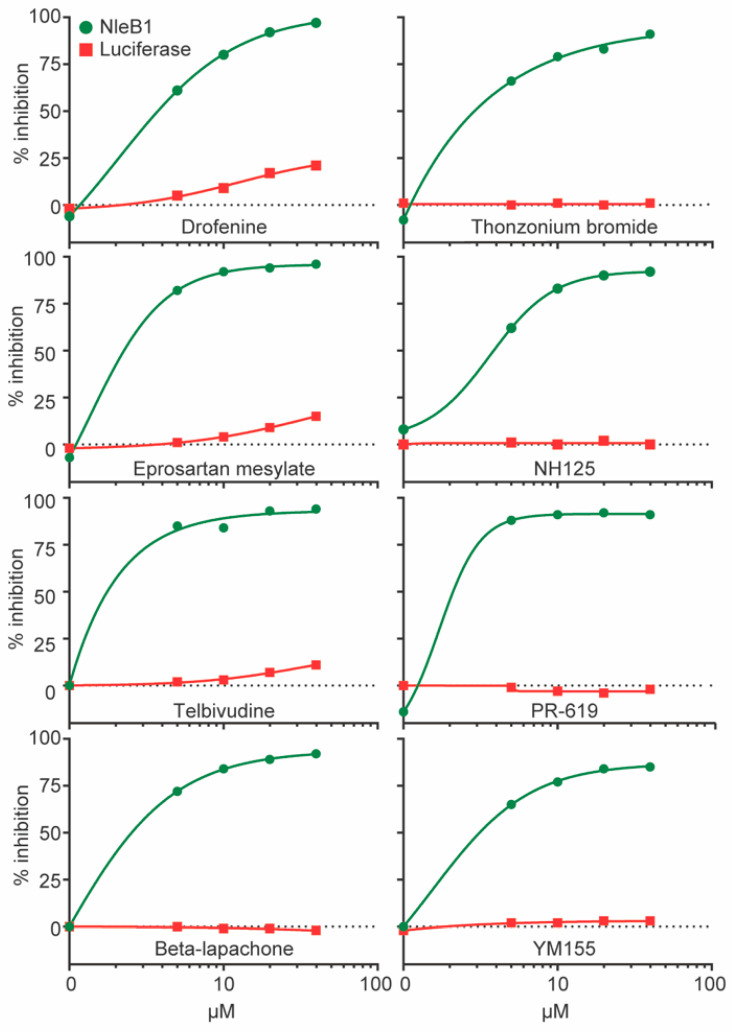
Reporter assay reconfirmation. NleB inhibition and luciferase inhibition assay results of eight primary targets from the Food and Drug Administration (FDA) and Bioactives libraries with IC_50s_ < 5 μM. Luciferase assays were conducted as described for HTS assays. NleB activity inhibition is plotted in green and luciferase activity inhibition is plotted in red, both as a function of individual compound concentrations from 0–40 µM.

We validated eight of the primary hits by performing additional reporter assays and determined that each of the tested compounds inhibited NleB activity without directly impacting the luciferase enzyme used in the primary assay (Figure 2). 

Thonzonium Bromide is a monocationic surface-active agent with surfactant and detergent properties used as an additive in ear and nasal drops [10]. Telbivudine is an antiviral drug used to treat hepatitis B infection [11]. Eprosartan mesylate is an angiotensin II receptor antagonist used to treat high blood pressure [12]. NH125 is a selective inhibitor of eukaryotic elongation factor 2 kinase with anti-bacterial properties [13]. Drofenine hydrochloride is a competitive inhibitor of butyrylcholinesterase [14]. Beta-Lapachone is a naturally occurring topoisomerase I inhibitor that induces apoptosis by inhibiting cell cycle progression [15]. PR-619 is a broad-range deubiquitylase inhibitor that induces ER stress [16].

**Figure 3:**
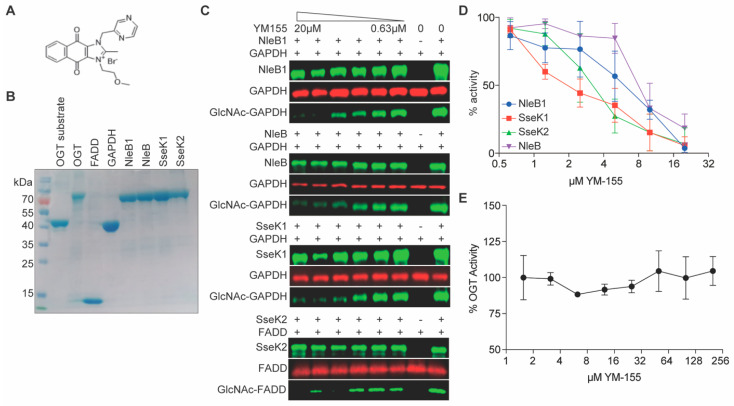
In vitro glycosylation assays. (**A**) YM155 structure. (**B**) SDS-PAGE analysis of recombinant proteins. (**C**) Western blot results for YM155 inhibition of NleB1, NleB, and SseK1 glycosylation of glyceraldehyde 3-phosphate dehydrogenase (GAPDH) and SseK2 glycosylation of Fas-associated protein with death domain (FADD). (**D**) Quantification of western blot signal intensities, n = 3. (**E**) UDP Glo Assay. O-linked N-acetylglucosaminyltransferase (OGT) activity as a function of [YM155], n = 3.

One of the new compounds we identified from this screen is sepantronium bromide (YM155; Figure 3A). YM155 is an inexpensive, commercially-available compound that has been previously characterized as a suppressor of survivin, a member of the inhibitor of apoptosis (IAP) gene family [17]. YM155 suppresses proliferation in a broad range of human cancer cell lines [18]. We investigated the activity of YM155 against NleB/SseK further. 

### 2.2. Glycosylation Assays

We tested the ability of YM155 to inhibit substrate glycosylation by the NleB/SseK enzymes. All recombinant enzymes (rNleB1, rNleB, rSseK1, and rSseK2) and substrates (rGAPDH and rFADD) were first purified using affinity chromatography (Figure 3B). YM155 inhibited EHEC NleB1, NleB, and SseK1 glycosylation of GAPDH in a concentration dependent manner (Figure 3C). YM155 also inhibited SseK2 glycosylation of the human FADD protein (Figure 3C). Western blot intensities were quantified as shown in Figure 3D.

We next tested whether YM155 also inhibited the human OGT enzyme, an essential serine/threonine N-acetylglucosamine (O-GlcNAc) transferase that maintains protein glycosylation homeostasis [19]. To do this, we performed a bioluminescence-based UDP-Glo glycosyltransferase assay to quantify OGT activity in presence of YM155. YM155 had no impact on OGT activity (Figure 3E), suggesting it is specific to the NleB/SseK Arg glycosyltransferases. 

### 2.3. Growth Assays

**Figure 4:**
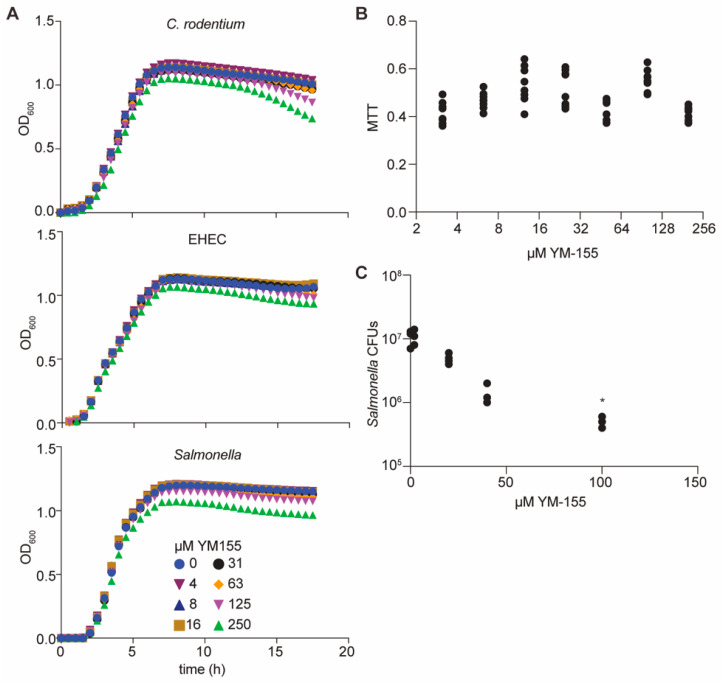
Cellular assays. (**A**) Bacterial growth assays. *C. Rodentium*, Enterohemorrhagic *E. coli* (EHEC), and *S. enterica* were cultured in LB media in the presence of YM155; bacterial growth was monitored as a function of time. (**B**) 3-(4,5-dimethylthiazol-2-yl)-2,5-diphenyltetrazolium bromide (MTT) assays. YM155 was added to RAW264.7 cells for 24 h and cell viability was assayed by monitoring MTT signal intensity. (**C**) *Salmonella* infection assays. RAW264.7 cells were seeded at 1 × 10^5^ cells/well in 24-well plates and YM155 was added 1 h before infection with 10^6^ CFUs of *Salmonella* for 30 min. * *p* < 0.05. Cells were incubated in medium containing 100 µg/mL gentamicin for 1 h, and then in 10 µg/mL gentamicin for an additional 23 h. Bacteria were released from RAW264.7 cells using 1% saponin, diluted in PBS, and plated for colony counts.

YM155 did not impact the growth rates of *C. rodentium*, EHEC, or *Salmonella enterica* when supplied at concentrations of up to 125 µM in bacterial cultures (Figure 4A). These data suggest that YM155 does not act as a general bacteriostatic or bactericidal agent. YM155 was not significantly toxic to mammalian cells, as inferred from 3-(4,5-dimethylthiazol-2-yl)-2,5-diphenyltetrazolium bromide (MTT) assay data (Figure 4B). These data suggested the potential use of YM155 as an anti-virulence small molecule. To determine whether YM155 might function in mammalian cells to reduce pathogen burdens, we quantified the impact of YM155 on *Salmonella* survival in cell culture. When YM155 was added to RAW264.7 cells prior to their infection with *Salmonella*, we observed that the number of *Salmonella* 24 h after infection was significantly reduced (Figure 4C). 

## 3. Discussion

We previously identified two small molecules, 100066N and 102644N, that inhibited the activities of NleB1, SseK1, and SseK2 [9]. In this study, we screened a larger library of 42,498 compounds and identified 200 other molecules that may have activity against NleB/SseK (Figure 1). We validated the activity of eight of these compounds in secondary screening assays (Figure 2). However, most of these compounds had unfavorable properties that precluded their further study. PR-619 exhibits cytotoxicity [20], and Beta-Lapachone causes DNA damage [21]. NH125 exhibited direct antimicrobial activity, whereas thonzonium bromide, telbivudine, eprosartan mesylate, and drofenine hydrochloride did not significantly reduce NleB1 activity when studied further in western blotting assays (data not shown). We therefore focused on YM155. 

YM155 was discovered as a small molecule inhibitor that suppresses survivin, a member of the inhibitor of apoptosis (IAP) family [17]. Survivin functions to inhibit caspase activation [22]. YM155 suppresses the proliferation of a broad range of human cancer cell lines [18]. YM155 also inhibits topoisomerase function and prevents double DNA strand repair [23].

Here, we determined that YM155 also functions as an NleB/SseK glycosyltransferase inhibitor. IC50s for the NleB/SseK orthologs were estimated at ~2 µM from luciferase reporter (Figure 2) and western blotting assays (Figure 3C,D). YM155 had no discernible toxicity in RAW264.7 cells, nor did it affect bacterial growth rates at concentrations less than 250 µM. Furthermore, YM155 had no apparent cross-reactivity with the mammalian OGT enzyme (Figure 3E). YM155 reduced the survival of *Salmonella* in RAW264.7 cells as a function of YM155 concentration (Figure 4C), suggesting its potential utility as an anti-virulence compound. Although YM155 appears to be less active than 100066N and 102644N [9], it has the advantage of being more soluble and amenable to chemical derivatization. 

While our primary goal is to discover, characterize, and use NleB/SseK inhibitors as anti-virulence therapeutics, it has not escaped our attention that an additional benefit of such inhibitors may be by synergizing with existing antibiotic regimens. Thus, future applications of our work would be to perform minimum inhibitory and minimal bactericidal concentration testing and quantify to what extent the NleB/SseK inhibitors may improve antibiotic efficacy. The future repurposing of previously characterized small molecules to prevent or treat bacterial infections could promote a medicinal chemistry campaign on NleB/SseK inhibitor development, as well as the development of other small molecules that inhibit effectors catalyzing unusual post-translational modifications, which could add impact to potential future studies. 

## 4. Materials and Methods

### 4.1. Cell Lines

Abelson-murine-leukemia-virus-induced, macrophage-like cells from BALB/c mice (RAW264.7) were purchased from ATCC and grown in Dulbecco’s Modified Eagle Medium (DMEM), supplemented with 10% fetal bovine serum (FBS) (Atlanta Biologicals, Minneapolis, MN, USA) and 100 µg/mL penicillin/streptomycin (Sigma, St. Louis, MO, USA) in 5% CO_2_. 

### 4.2. Library Screening

HTS assays were performed similarly to as described previously [9] in 50 mM Tris-HCL buffer pH 7.5, 100 mM NaCl, 1 mM DTT, 10 mM MgCl_2,_ and 0.5% DMSO. rNleB1 (150 nM) was pre-incubated for 30 min in 384 well-microplates, containing compounds (20 µM) transferred acoustically using ECHO555 (Labcyte Inc, Indianapolis, IN, USA). Luminescence was read 2 h after the addition of the UDP detection reagent. The percent inhibition was normalized to the controls and compounds that inhibited the assay to >3 standard deviations plus the plate median were identified as hits.

### 4.3. Protein Purification

Protein purification was performed similarly to as described previously [24]. *E. coli* BL21 (DE3) strains harboring pET42a-, pET28a-, and pET15b-expression plasmids were grown at 37 °C in LB until an OD600 of 0.4, at which time 0.5 mM IPTG was added for 4 h. Cells were centrifuged, and the pellet was resuspended in lysis buffer (50 mM sodium phosphate buffer pH 8.0, 0.5 mg/mL lysozyme). The suspension was incubated on ice for 30 min with occasional shaking and then treated with 50 mM sodium phosphate buffer pH 8.0, 2 M NaCl, 8 mM imidazole, 20% glycerol, 2% Triton X-100, for 30 min. Cell lysates were sonicated and centrifuged, and then the resultant supernatant was added to 2 mL Ni-NTA resin (Qiagen, Germantown, MD, USA) for 1 h of rotation at 4 °C. The mixture was loaded on a Poly-Prep Chromatography Column (Bio-Rad Hercules, CA, USA) and washed with 10 mL of 50 mM sodium phosphate buffer pH 8.0, 600 mM NaCl, 60 mM imidazole, 10% glycerol. Proteins were eluted in 2 mL 50 mM sodium phosphate buffer pH 8.0, 600 mM NaCl, 250 mM imidazole, and 10% glycerol, and dialyzed into the same buffer lacking imidazole. 

### 4.4. Glycosylation Assays

In vitro glycosylation assays were performed as described previously [24]. Enzymes (200 nM of NleB1, NleB, SseK1, or SseK2) were incubated with 1 µM of GAPDH or FADD +/− serial dilutions of YM155 in 50 mM Tris-HCl buffer pH 7.4, 1 mM UDP-GlcNAc, 10 mM MnCl_2_, and 1 mM DTT. After 2 h incubation at RT, samples were subjected to western blotting using anti-R-GlcNAc and anti-His tag monoclonal antibodies (Abcam, Cambridge, MA, USA). Signal intensities were quantified using LI-COR Image Studio software (LI-COR Biosciences, Lincoln, NY, USA), and inhibition was calculated by quantifying the relative reduction in substrate glycosylation.

### 4.5. OGT Assays

UDP-Glo luminescence assays were performed to determine whether YM155 inhibits the mammalian OGT enzyme. Reactions were performed in 96 well-microplates following manufacturer’s instructions with the UDP-Glo™ Glycosyltransferase Assay Kit (Promega). Reactions contained 200 nM OGT in 25 mM Tris-HCL buffer pH 7.5, 12.5 mM MgCl_2_, 0.06 mg/mL BSA, 1 mM DTT, 50 μM OGT peptide substrate, and 100 μM UDP-GlcNAc, and included YM155 from 1–200 μM. Reactions were incubated for 1 h at 22 °C, and luminescence signals were quantified using a FLUO star microplate reader (BMG Labtech, Cary, NC, USA).

### 4.6. MTT Assays

MTT assays were performed as specified by Millipore using RAW264.7 cells in the presence of 2-fold serial dilutions of YM155. Formazan absorbance was measured at 570 nm using an Epoch Microplate Spectrophotometer (BioTek, Winooski, VT, USA).

### 4.7. Bacterial Growth Assays

Bacterial cultures were grown overnight, diluted 1:200 in LB in the presence of 2-fold serial dilutions of YM155 (0–250 µM), and then grown at 37 °C for 18 h. Bacterial growth was monitored by measuring the absorbance of the culture medium at OD_600_.

### 4.8. Macrophage Infection Assays

RAW264.7 cells were seeded at 1 × 10^5^ cells/well in 24-well plates, and YM155 was added 1 h before infection. Bacterial cultures were grown overnight, and 10^6^ CFUs were added to each well for 30 min. Cells were treated with 100 µg/mL gentamicin for 1 h and then with 10 µg/mL gentamicin for an additional 23 h. Bacteria were released from RAW264.7 cells using 1% saponin (Sigma), diluted in PBS, and plated for colony counts.

## Data Availability

All relevant data are contained within the article.
